# The Prince of Wales's Hospital Fund

**Published:** 1901-11-16

**Authors:** 


### The Institutional Workshop.

### THE PRINCE OF WALES'S HOSPITAL FUND.

### THE CORONATION GIFT.

It is by no means an easy matter to select an acceptable gift for Royal personages. Owing to the close identification of the King with the Fund which he himself founded, the difficulty of selecting a Coronation gift from the nation has on the present occasion been happily solved. By helping the Fund to remove the financial embarrassment of the hospitals the nation will be helping the King to carry out a great wish of his heart.

It is for these reasons that the appeal with which the general public is now familiar has been sent out, and which, as we stated last week, has already been responded to by companies, employees, and individuals to the amount of £30. Such an appeal demonstrates the fact once more that though statistics may appeal to the few, it is the thought of human suffering alleviated—or awaiting alleviation—which appeals to the many.

A glance through the accounts of the Prince of Wales's Hospital Fund for London, since its foundation rather more than four years ago, shows that it has been the means of getting together the sum of £366,908, an average of nearly £92,000 for each year, taking it only up to the end of 1900. This sum has been, roughly speaking, divided into two parts; half has been awarded to various hospitals and convalescent homes, while the other half has been invested in the interests of the future.

The actual sum which has been apportioned in grants is £181,326; £96,350 in annual grants, £81,976 in special grants, and £3,000 to convalescent homes. Some eighty-five voluntary hospitals in and about the Metropolis have been visited by the representatives of the Fund, besides those which have not applied for an annual grant, and fifteen convalescent homes are being helped. The working expenses for the four years have amounted to over £3,000, more than £1,000 being spent in 1900 alone in correspondence, appeals, lists of contributors, circulars, etc.

It will be remembered that the idea of the Royal founder of this Fund was to help the already existing hospitals, rather than to build new ones; and the assistance that has been given has been bestowed at an infinitely less cost than would have been the case had the method of building new institutions been the one selected. The Fund found hundreds of beds closed for want of money; by means of the abovementioned awards it has enabled nearly three hundred of these to be re-opened, thus providing for the in-treatment of an average of 1,000 additional patients for each year of its existence. This is reckoned on the usually accepted average of fourteen patients to each bed per year.

Besides the actual re-opening of beds, the hospitals have been regularly visited by the committee appointed for the purpose, who have urged extensions, pointed out defects, made special grants towards capital expenditure, set a high standard of administration before boards of management, and enlarged the area of public sympathy.

About 450 beds are still stated to be closed, half the number being absolutely unused wards; these could be immediately opened for the reception of some of the most pressing cases awaiting admission, if the necessary means were at hand. Moreover, new methods, new buildings, reconstruction to meet the requirements of modern surgical and medical science, all add to the heavy current expenses of the hospitals, making, with the constant growth of population, a serious addition to the responsibilities of management boards.

It may be added that His Majesty the King, who was until lately President of the Fund, has now become its Patron, while H.R.H. George, Prince of Wales, has accepted the position of President.

